# Thermal Conductivity and High-Frequency Dielectric Properties of Pressureless Sintered SiC-AlN Multiphase Ceramics

**DOI:** 10.3390/ma11060969

**Published:** 2018-06-08

**Authors:** Jialin Gu, Lingling Sang, Bo Pan, Yongbao Feng, Jian Yang, Xiaoyun Li

**Affiliations:** State Key Laboratory of Materials-Oriented Chemical Engineering, College of Materials Science and Engineering, Nanjing Tech University, Nanjing 210009, China; gujialin8250@163.com (J.G.); 15951607063@163.com (L.S.); panbo1220@163.com (B.P.); fyb_njut@163.com (Y.F.); yangjian1976@163.com (J.Y.)

**Keywords:** SiC-AlN, pressureless sintering, thermal conductivity, dielectric properties

## Abstract

SiC-AlN multiphase ceramics with 10 wt. %Y_2_O_3_-BaO-SiO_2_ additives were fabricated by pressureless sintering in a nitrogen atmosphere. The effects of SiC contents and sintering temperatures on the sinterability, microstructure, thermal conductivity and high-frequency dielectric properties were characterized. In addition to 6H-SiC and AlN, the samples also contained Y_3_Al_5_O_12_ and Y_4_Al_2_O_9_. SiC-AlN ceramics sintered with 50 wt. % SiC at 2173 K exhibited the best thermal diffusivity and thermal conductivity (26.21 mm^2^·s^−1^ and 61.02 W·m^−1^·K^−1^, respectively). The dielectric constant and dielectric loss of the sample sintered with 50 wt. % SiC and 2123 K were 33–37 and 0.4–0.5 at 12.4–18 GHz. The dielectric constant and dielectric loss of the samples decreased as the frequency of electromagnetic waves increased from 12.4–18 GHz. The dielectric thermal conductivity properties of the SiC-AlN samples are discussed.

## 1. Introduction

Silicon carbide (SiC) is a promising ceramic material due to its excellent physical-chemical properties, including good mechanical properties at room and high temperatures, high thermal conductivity, high hardness, good dielectric properties, and excellent resistance to corrosion and oxidation [1,2,3,4]. Thus, SiC ceramic can be used in a wide range of industrial and electronical applications such as the photovoltaic and semiconductor industry, structural materials, high-temperature materials, abrasives and traveling-wave tube [5,6,7]. However, due to its strong Si–C covalent bands, high-purity SiC is very difficult to sinter to be fully dense at a low temperature without sintering additives or external pressure [8].

As well as its excellent mechanical properties and thermal conductivity, SiC is also an superior microwave absorbent. SiC and other substrates can form fine microwave attenuation composites which can be applied as microwave absorbers in traveling wave tubes and electron accelerators to suppress parasitic electromagnetic oscillations and damp higher order modes [9,10,11]. Trends in the electronic components manufacturing and applications result in an urgent desire for high thermal conductive microwave attenuation composites. BeO-based attenuation composite is a kind of excellent material which has high thermal conductivity, but its application is limited due to the toxicity of BeO [12]. The latest study demonstrates that Aluminum nitride (AlN) is an interesting substrate for microwave attenuation composites because of its high thermal conductivity, low density (3.26 g·cm^−3^), high electrical resistivity and thermal expansion coefficient. Those properties, along with its nontoxic characteristics, make AlN a popular substrate material instead of BeO. AlN and SiC also can form a solid solution under certain conditions [13], which can largely improve the sintering activity, mechanical properties, electrical conductivity, and oxidation resistance of materials [14,15,16].

It is well known that it is difficult for AlN and SiC to achieve high density due to their strong covalent bands. For densification, rare-earth oxides and metallic oxides are often added as sintering additives to fabricate AlN and SiC ceramics. At present, SiC-AlN multiphase ceramics usually are made by hot pressing. However, the hot press sintering has a high cost, long cycle, and low efficiency, and pressureless sintering has gradually become the focus of recent research. However, SiC-AlN multiphase ceramics prepared under the pressureless sintering process condition often require higher sintering temperatures [17]. Therefore, it is necessary to find suitable sintering additives to reduce the sintering temperature through liquid-phase sintering and mass transfer to obtain dense SiC-AlN multiphase materials.

In this paper, SiC-AlN multiphase ceramics were fabricated by pressureless sintering, in which we chose Y_2_O_3_-BaO-SiO_2_ as sintering additives. Then thermal conductivities and high-frequency dielectric properties were investigated and discussed. The selection of sintering additives refers to the additives used in hot-press sintering, Y_2_O_3_ is a suitable sintering additive for AlN ceramics. The additives for the preparation of dense SiC ceramics by hot-press sintering are MgO-Al_2_O_3_-SiO_2_, Y_2_O_3_ (or other rare earth oxides)-Al_2_O_3_-SiO_2_. According to the phase diagram of BaO-SiO_2_ and MgO-SiO_2_ [18], the lowest temperature of the liquid phase occurs in the MgO-SiO_2_ system is around 1823 K. It is significantly higher than that of BaO-SiO_2_ system. When the molar ratio of BaO:SiO_2_ is 1:2, the liquid phase temperature is 1696 K. All are liquid phases at 1719 K, which is about 100 K lower than the MgO-SiO_2_ system. Therefore, the sintering additives of BaO-SiO_2_ system is worth exploring.

## 2. Materials and Methods

The particle size and some specific parameters of raw materials were designed and are given in Table 1. To prepare SiC-AlN multiphase ceramics with Y_2_O_3_-BaO-SiO_2_ additives, α-SiC, AlN, Y_2_O_3_, 1.12 wt. % BaO and 0.88 wt. % SiO_2_ (BaO:SiO_2_ were 1:2) were mixed by ball milling in alcohol for 6 h in a polypropylene jar at a speed of 180 r/min. Then the slurry mixture was dried, crushed and sieved (140 mesh). Discs with diameter of 27 mm and thickness of 5 mm were prepared by uniaxial pressing at 100 MPa followed by cold isostatic pressing at 300 MPa. Then pressureless sintering was performed at temperatures from 2023 to 2173 K for 1 h in a flowing nitrogen atmosphere. Three series of different Y_2_O_3_ contents (DY, 4–8 wt. %), temperatures (DT, 2023 K, 2073 K, 2123 K, 2173 K) and different SiC contents (DS, 40 wt. %, 45 wt. %, 50 wt. %, 55 wt. %, 60 wt. %) were processed, where the temperature in DY was 2123 K and 50 wt. % SiC, the SiC content of DT was 50 wt. %, and the temperature in DS was 2123 K respectively.

The density and apparent porosity of samples was determined by the Archimedes method. The theoretical density ρ_th_ was calculated from the law of mixtures and was not corrected for weight loss during sintering. The calculation method is shown in Equation (1):

(1) $$\rho_{\mathrm{th}}=\sum_{i=1}^{n}P_{i}\times \rho_{i}$$

where P_i_ is the volume fraction of each component in the multiphase ceramic, ρ_i_ is the theoretical density of the corresponding component. Equation (2) of the calculation formula of relative density ρ_re_.

(2) $$\rho_{\mathrm{re}}=\frac{\rho }{\rho_{\mathrm{th}}}$$

X-ray diffraction (XRD, Rigaku SmartLab, Tokyo, Japan) with CuK_α_ was used to characterize the crystalline phase of samples. The morphology of fracture surface was observed by scanning electron microscopy (SEM, JSM-5900, JEOL, Tokyo, Japan). Thermal diffusivity (α) was measured by flash method (LFA447, Netzsch, Selbu, Germany). The samples which were used to measure thermal diffusivity at 298 K were processed as disks (Ø12.7 mm × 2 mm). In addition, the experiment was conducted three times in the same conditions, then an average value of three measurements was taken. The thermal conductivity (λ) was calculated according the formula:

(3) $$\lambda ={\;\alpha \;\rho \;C}_{p}$$

where ρ is the density and C_P_ is heat capacity of specimen. High-frequency dielectric properties of SiC-AlN ceramics were determined by a translation/reflection method using PNA-N5244A network analyzer (Agilent, Polo Alto, CA, USA). Samples of SiC-AlN ceramics were processed into 15.80 mm × 7.90 mm × 2 mm, using a rectangular waveguide transmission line as a fixture. Then, the Agilent PNA-N5244A network analyzer was used to test its complex permittivity in Ku-band (12.4–18 GHz), including dielectric constant and dielectric loss.

## 3. Results and Discussion

**Figure 1:**
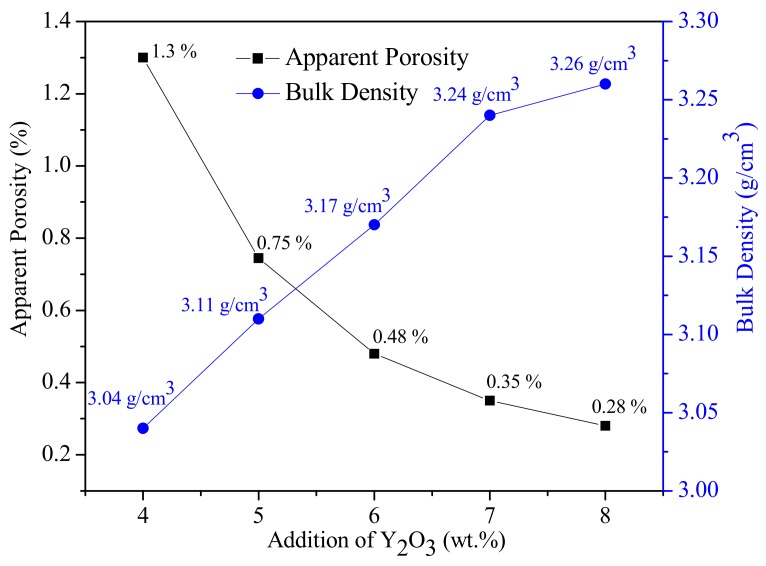
Apparent porosity and bulk density of 50 wt. %SiC-40 wt. %AlN multiphase ceramics with 4–8 wt. %Y_2_O_3_-1.12 wt. %BaO-0.88 wt. %SiO_2_ sintering additive.

The change of apparent porosity and bulk density of the 50 wt. %SiC-40 wt. %AlN multiphase ceramics with 4–8 wt. %Y_2_O_3_-1.12 wt. %BaO-0.88 wt. %SiO_2_ sintering additive at 2073 K for 1 h are shown in Figure 1. As can be seen that with the content of Y_2_O_3_ increased, the apparent porosity of the SiC-AlN multiphase ceramics decreased, and the bulk density gradually increased. When 4 wt. % of Y_2_O_3_ was added, the density of the sample was poor, and the bulk density was only 3.04 g/cm^3^. With the content of Y_2_O_3_ was up to 8 wt. %, the apparent porosity of the material was obviously reduced, and the density was increased. When the content of Y_2_O_3_ was increased to 8 wt. %, the porosity rate reached a minimum of 0.28%, the tendency of decreasing the apparent porosity was slow down, and the bulk density changed little, the material is substantially dense. It suggests that this sintering additives were proper for the densification of SiC-AlN ceramics.

**Figure 2:**
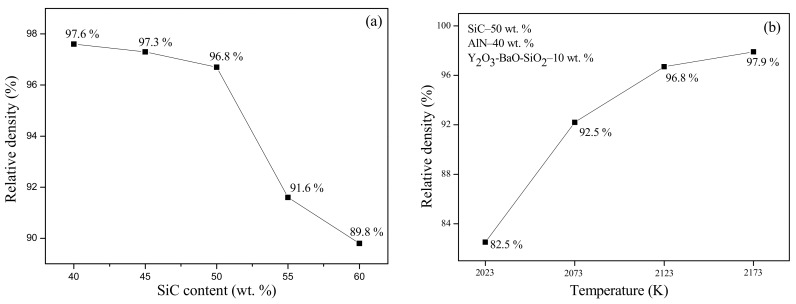
Relative density of the SiC-AlN ceramics. (**a**) Different SiC contents; (**b**) different sintering temperatures.

Pressureless sintering at 2123 K for 1 h, the relative density of SiC-AlN ceramics with different SiC contents are shown in Figure 2a. Relative density of the pressureless sintering samples ranged from 97.6% to 89.8% as the content of SiC increased. When the content of SiC increases from 40% to 50%, the relative density gradually decreases from 97.6% to 96.8%, while increasing the content of SiC to 60%, the relative density rapidly drops to 89.8%. The samples cannot be sintered densely while the content of SiC was up to 60%, but when the sintering temperature was less than 2123 K the sample of 50 wt. %SiC-40 wt. %AlN-10 wt. %Y_2_O_3_-BaO-SiO_2_ additives at different temperatures of pressureless sintering for 1 hour, the relationship of relative density and sintering temperature are shown in Figure 2b. When the sintering temperatures went up, the relative densities ranged from 82.5% to 97.9% (Figure 2b). This illustrates that SiC content and sintering temperature are two of main factors which influence the sintering property of SiC-AlN multiphase ceramics. There is not enough energy to accelerate mass transport when the content of SiC is too much or the sintering temperature is too low. Except for these two factors, additives content, holding time, and pressure also will affect the densification of the multiphase ceramics. 

**Figure 3:**
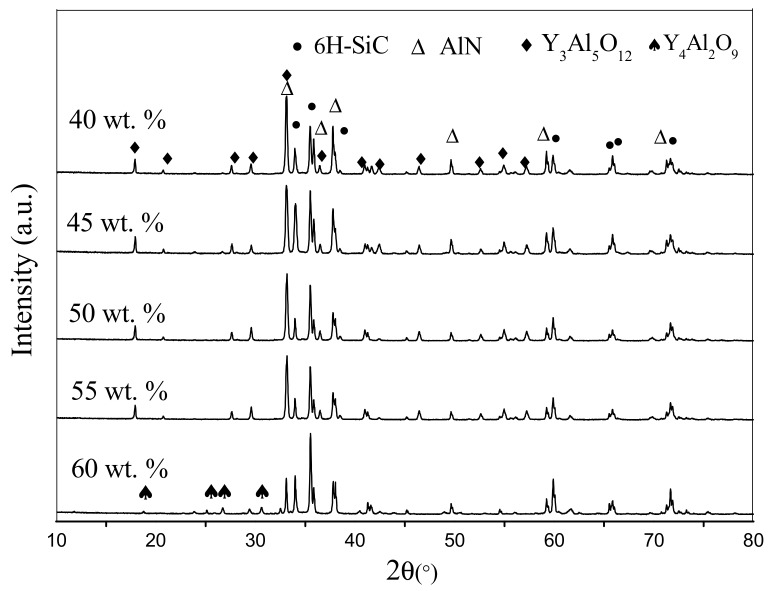
XRD patterns of SiC-AlN ceramics with different SiC contents.

Figure 3 exhibits the XRD pattern of the SiC-AlN multiphase ceramics with different SiC contents. 6H-SiC and AlN are the main phases of samples, and the secondary phase is Y_3_Al_5_O_12_ (YAG). The main phases of the materials are not influenced by the different contents of SiC. In the present samples, Y_2_O_3_ additive reacted with Al_2_O_3_, which is always present on the surface of the AlN particle, to form YAG. BaO and SiO_2_ would form eutectic mixtures as amorphous materials. As the content of SiC increased to 60 wt. %, the Al_2_O_3_ brought by AlN largely decreased. Then the excessive Y_2_O_3_ reacted with YAG to form Y_4_Al_2_O_9_. All the materials formed by sintering additives owned low melting point so that can exist in grain boundary as liquid phase. The liquid phase was responsible for the densification of the samples by liquid-phase sintering [19].

**Figure 4:**
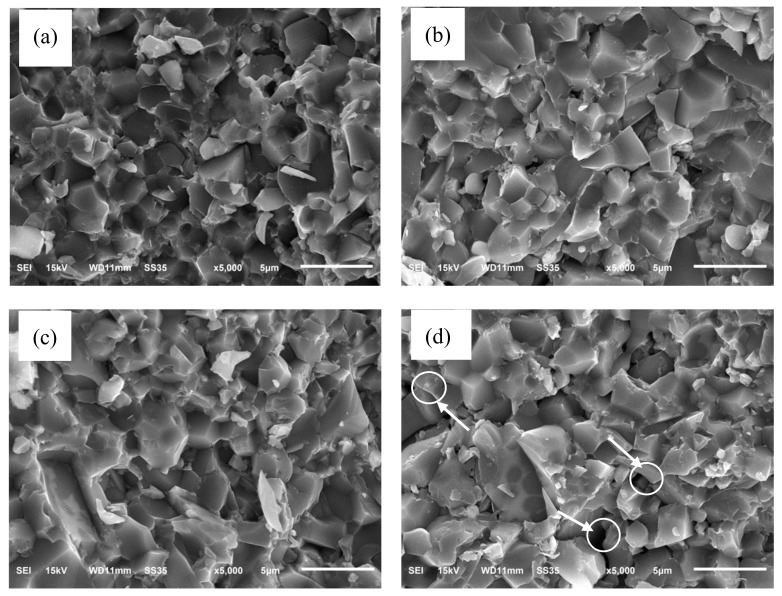

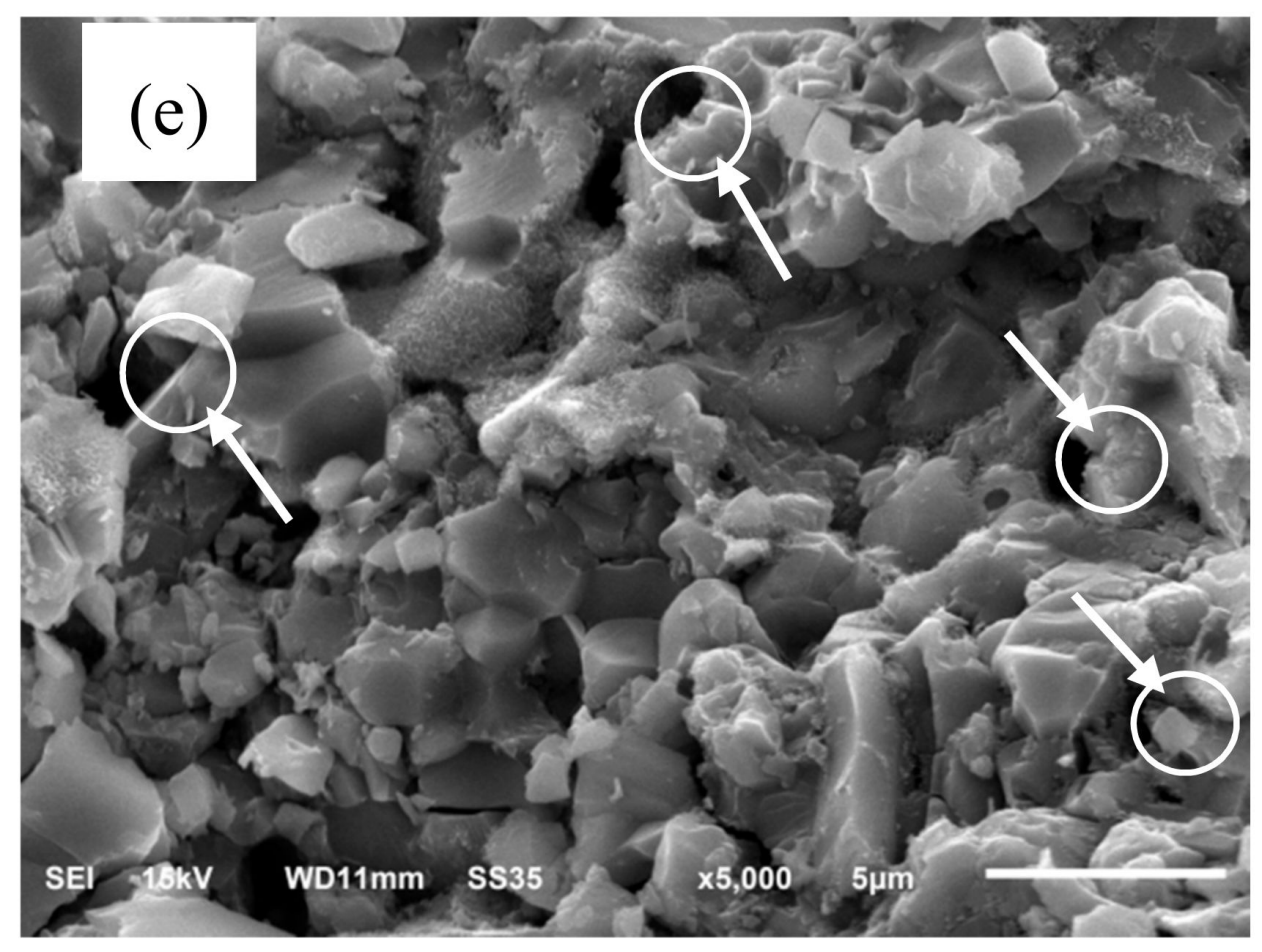
SEM images of the SiC-AlN ceramics with different SiC contents: (**a**) 40 wt. %; (**b**) 45 wt. %; (**c**) 50 wt. %; (**d**) 55 wt. %; (**e**) 60 wt. %. Circles and arrows mark the pore region.

Figure 4 shows typical fracture surfaces of the SiC-AlN ceramics with different SiC contents. Except the sample with 55 wt. % and 60 wt. % SiC, all the other samples exhibited a dense microstructure with little pores. The area indicated by the circles and arrows in Figure 4 is the pore region, which is in excellent agreement with the result of relative density. Since the self-diffusion coefficient of SiC is lower than that of AlN, as the content of SiC increased, the pores of SiC-AlN ceramics increased. The main fracture mode of the dense samples was intergranular fracture. The grain of sample with 40 wt. % SiC was more uniform than others and its size was about 3 µm. The solid vaporization pressures of AlN far exceeds that of SiC powder, so the evaporation rate of AlN was faster than that of SiC when SiC-AlN ceramics were sintered [20]. AlN firstly vaporized and then deposited around the SiC particles to prevent the exaggerated growth of SiC grains during the sintering process. As the content of SiC increased, the content of AlN in samples became less so that it is not enough to prevent the growth of grains. It can be seen that more and more big size grains occurred in the samples with the content of SiC increasing in Figure 4.

**Figure 5:**
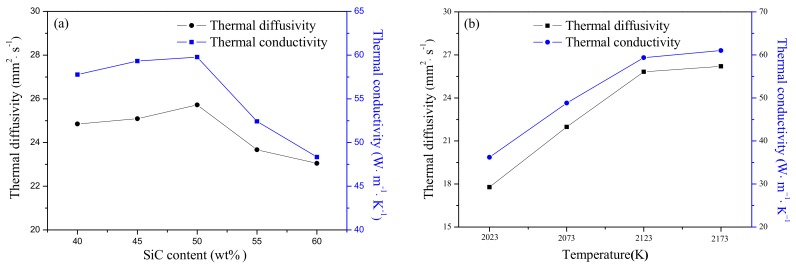
Thermal properties of SiC-AlN ceramics: (**a**) different SiC contents; (**b**) different sintering temperature.

Figure 5 exhibits the thermal properties of SiC-AlN samples at room temperature with different conditions. The content of SiC increased from 40 to 50 wt. %, and the thermal conductivity increased slowly. It is speculated that the main reason is the grain boundary phase formed by the sintering additive is more beneficial for reducing phonon scattering between grains. The main reason for the decrease of the thermal conductivity of 55–60 wt. % SiC samples is the presence of the pores. The highest value of thermal diffusivity was 25.72 mm^2^·s^−1^ when the content of SiC was 50 wt. %, but it reduced gradually with increasing SiC content. As with the thermal diffusivity, the thermal conductivity reached a peak of 59.78 W·m^−1^·K^−1^ for the sample of 50 wt. % SiC (Figure 5a). Both the thermal diffusivity and thermal conductivity went up with increasing sintering temperature to 26.21 mm^2^·s^−1^ and 61.02 W·m^−1^·K^−1^, respectively. Polycrystalline SiC ceramics and polycrystalline AlN ceramics all have high thermal conductivity (270 W·m^−1^·K^−1^ and 200 W·m^−1^·K^−1^, respectively.) [21,22]. However, it had a large difference with the experimental values which achieved by pressureless sintering SiC-AlN ceramics, as shown in Figure 5. The large difference between monophasic ceramics and multiphase ceramics was attributed to the increased phonon scattering in SiC-AlN ceramics. The main reason was that the formation of SiC-AlN solid solution which largely increase phonon scattering to decrease the thermal conductivity of samples. In addition, the presence of a grain boundary and grain defects in samples also would increase phonon scattering [23,24,25] The phenomenon that grain size grew up with increasing SiC content can be seen in Figure 4, which will decrease phonon scattering. Thus, the thermal conductivity increased as the increase of SiC content. When SiC content exceeded 50 wt. %, the thermal conductivity sharply decreased due to the existence of porosity. It suggests that the presence of pores reduces thermal conductivity. As the sintering temperature was below 2123 K, the thermal conductivity increased obviously because of the decrease of residual porosity. When exceeding 2123 K, the thermal conductivity of fully dense samples increased slightly, which was mainly affected by the growth of grains in samples. Except for forming a solid solution, the main factor affecting the thermal conductivity in this experiment is the scattering of phonons by grain boundary phases and pores, and the dense samples are mainly affected by the scattering of the grain boundary phase, samples with large porosity have mainly stomatal scattering.

**Figure 6:**
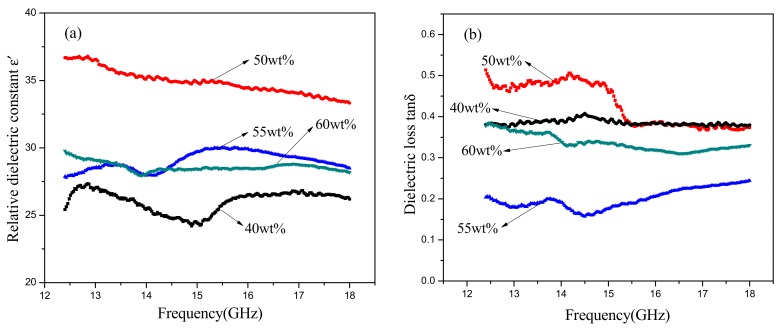
Dielectric properties of SiC-AlN ceramics with different SiC contents at high frequencies: (**a**) relative dielectric constant; (**b**) dielectric loss.

The effect of SiC content on the dielectric properties (12.4–18GHz) of SiC-AlN ceramics is shown in Figure 6. It can be seen that both the dielectric constant (ε′) and dielectric loss (tan δ) went up to the maximum values (33–37 and 0.4–0.5, respectively) at 50 wt. % SiC and then decreased with increasing SiC content. When SiC content exceeded 55 wt. %, the dielectric properties would decrease due to the existence of porosity. The material factors determining the energy loss in a dielectric is loss factor (ε″) which is decided by the product of the dielectric constant and the dielectric loss. When the content of SiC was 50 wt. %, the energy loss of the samples reached the largest and the microwave of this frequency could be attenuated well. As the frequency increased, the dielectric constant gradually decreased, but the dielectric loss went up to a summit at 14 GHz and then reduced. It can be inferred that the maximum dielectric loss occurred when the period of relaxation process may be the same as the period of the applied field. SiC-AlN multiphase ceramics exhibit space charge polarization resulting from different electrical conductivity between SiC and AlN. The motion of the charge carriers occurs easily through one phase but is interrupted when it reaches a phase boundary. This causes a buildup of charges at the interface which corresponds to a large polarization and high dielectric constant and dielectric loss [26]. However, a long time for space charge to establish makes it difficult to form space charge polarization. Dipole polarization losses also exist in the samples because of the formation of SiC-AlN solid solution. An Al atom has three valence electrons, but an Si atom has four. When an Al atom dissolves into SiC lattice to replace an Si atom, the compound will capture an electron and form an electropositive hole. As an electromagnetic field is applied and changes, the electronegative Al atoms and the electropositive holes can be considered to be a pair of dipoles, and dipole polarization occurs [16]. In addition, the defects from solid solution also contribute to dielectric properties of SiC-AlN ceramics. The dielectric constant and dielectric loss of SiC-AlN ceramics are mainly caused by conductivity loss and dipole polarization loss. Therefore, the dielectric properties of dense samples increased when dipole polarization and solid solution increased with the rise of SiC content.

**Figure 7:**
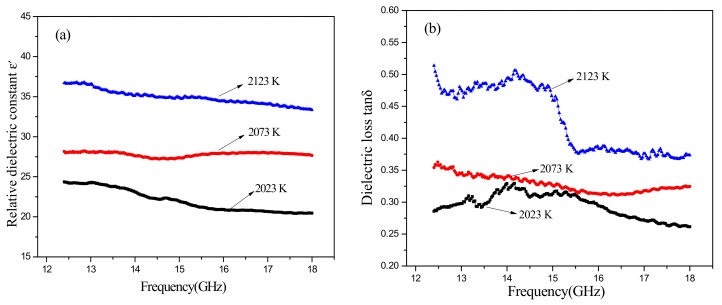
Dielectric properties of SiC-AlN ceramics with different sintering temperature at high frequencies: (**a**) relative dielectric constant; (**b**) dielectric loss.

Figure 7 illustrates the dielectric properties of SiC-AlN ceramics with different sintering temperature. The dielectric constant and the dielectric loss rose as the sintering temperature increased. However, the opposite trend was shown with the increase of frequency. Similarly, with Figure 6b, it finds a dielectric loss peak at 14 GHz in Figure 6b. It can infer that what affects the dielectric properties of samples is mainly porosity when sintering temperature varied. 

## 4. Conclusions

(1) Fully dense SiC-AlN multiphase ceramics were prepared by pressureless sintering in a nitrogen atmosphere, in which Y_2_O_3_-BaO-SiO_2_ were used as additives. Y_3_Al_5_O_12_ and Y_4_Al_2_O_9_ existed as minor phases in the samples.
(2) SiC-AlN multiphase ceramics sintered with the content of 50 wt. % SiC at 2173 K showed the maximum thermal diffusivity and thermal conductivity (26.21 mm^2^·s^−1^ and 61.02 W·m^−1^·K^−1^). The high thermal conductivity of the samples was attributed to densification microstructure and rare porosities which would decrease phonon scattering.
(3) The main factors that affect the microwave attenuation of the SiC-AlN multiphase ceramics were conductivity loss and dipole polarization loss. In addition, various defects caused by solid solution also contributed to the dielectric loss of the samples. When the content of SiC was 50 wt. % and temperature was 2123 K, the dielectric constant and dielectric loss of SiC-AlN multiphase ceramics achieved the highest values (33–37 and 0.4–0.5, respectively) at high frequency waveband.

## Figures and Tables

**Table 1:** Characteristics of the raw materials.

Raw Materials	Chemical Formula	Purity (wt. %)	Average Particle size (µm)	Source
α-Silicon carbide	α-SiC	≥98%	~0.5	Pingdingshan Yicheng Co., Ltd. China
Aluminum nitride	AlN	≥99%	~0.6	Tokuyomo Co., Ltd. Tokyo, Japan
Yttrium oxide	Y_2_O_3_	99.9%	<5	Yuelong Chemical Co., Ltd. Shanghai, China
Barium oxide	BaCO_3_	99.9%	<6	Sinopharm Group Co., Ltd. Shanghai, China
Silicon oxide	SiO_2_	99.9%	<5	Sinopharm Group Co., Ltd. Shanghai, China

## References

[1] Onbattuvelli V.P., Enneti R.K., Atre S.V. (2012). The effects of nanoparticle addition on the densification and properties of SiC. Ceram. Int..

[2] Kumar B.M, Kim Y.W., Lim D.S., Seo W.S. (2011). Influence of small amount of sintering additives on unlubricated sliding wear properties of SiC ceramics. Ceram. Int..

[3] Lim K.Y., Kim Y.W., Kim K.J. (2014). Electrical properties of SiC ceramics sintered with 0.5wt% AlN-Re_2_O_3_ (Re = Y, Nd, Lu). Ceram. Int..

[4] Zhang J.X, Jiang D.L., Lin Q.L., Chen Z.G., Huang Z.G. (2013). Gelcasting and pressureless sintering of silicon carbide ceramics using Al_2_O_3_-Y_2_O_3_ as the sintering additives. J. Eur. Ceram. Soc..

[5] Hayun S., Paris V., Mitrani R., Kalabukhov S., Dariel M.P., Zaretsky E., Frage N. (2012). Microstructure and mechanical properties of silicon carbide processed by spark plasma sintering(SPS). Ceram. Int..

[6] Liang H.Q., Yao X.M., Zhang H., Liu X.J., Huang Z.G. (2014). In situ toughening of pressureless liquid phase sintered alpha-SiC by using TiO_2_. Ceram. Int..

[7] Lim K.Y., Kim Y.W., Nishimura T., Seo W.S. (2013). High temperature strength of silicon carbide sintered with 1 wt. % aluminum nitride and lutetium oxide. J. Eur. Ceram. Soc..

[8] Magnani G., Sico G., Brentari A., Fabbri P. (2014). Solid-state pressureless sintering of silicon carbide below 2000 °C. J. Eur. Ceram. Soc..

[9] Calame J.P., Abe D K., Levush B., Danly B.G. (2001). Variable temperature measurements of the complex dielectric permitivity of lossy AlN-SiC composites from 26.5–40 GHz. J Appl. Phys..

[10] Zhang X.Y., Tan S.H., Zhang J.X., Jiang D.L. (2004). Lossy AlN-SiC composites fabricated by spark plasma sintering. J. Mater. Res..

[11] Kobayashi R., Tatami J., Wakihara T., Komeya K., Meguro T., Tu R., Goto T. (2010). Evaluation of grain-boundary conduction of dense AlN-SiC solid solution by scanning nonlinear dielectric microscopy. J. Am. Ceram. Soc..

[12] Mikijelj B., Abe D.K., Hutcheon R. (2003). AlN-based lossy ceramics for high average power microwave devices: Performance-property correlation. J. Eur. Ceram. Soc..

[13] Cutler I.B., Miller P.D., Rafaniello W., Park H.K., Thompson D.P., Jack K.H. (1978). New materials in the Si-C-Al-O-N and related systems. Nature.

[14] Lim K.Y., Kim Y.W., Kim K.J. (2014). Mechanical properties of electrically conductive silicon carbide ceramics. Ceram. Int..

[15] Chu A., Qin M., Rafi-ud-din, Dong Y.H., Guo S.B., Qu X.H. (2015). Two-step carbothermal synthesis of AlN-SiC solid solution powder using combustion synthesized precursor. J. Am. Ceram. Soc..

[16] Gao P., Jia C.C., Cao W.B., Wang C.C., Liang D., Xu G.L. (2014). Dielectric properties of spark plasma sintered AlN/SiC composite ceramics. Int. J. Miner. Metall. Mater..

[17] Li J.F., Watannabe R. (1994). Pressureless sintering and high-temperature strength of SiC-AlN ceramics. Ceram. Soc. Jpn..

[18] Osborn E.F., Muan A. (1964). Phase Diagrams for Ceramists.

[19] Liang H.Q., Yao X.M., Zhang J.X., Liu X.J., Huang Z.G. (2014). The effect of rare earth oxides on the pressureless liquid phase sintering of α-SiC. J. Eur. Ceram. Soc..

[20] Pan Y., Tan S., Jiang D. (1999). In-situ characterization of SiC-AlN multiphase ceramics. J Mater. Sci..

[21] Virkar A.V., Jackson T.B., Culter R.A. (1989). Thermodynamic and kinetic effects of oxygen removal on the thermal conductivity of aluminum nitride. J. Am. Ceram. Soc..

[22] Nakano H., Watari K., Kinemuchi Y., Ishizaki K., Urabe K. (2004). Microstructural characterization of high-thermal-conductivity SiC ceramics. J. Eur. Ceram Soc..

[23] Bentsen L.D., Haselman D.P.H., Ruh R. (1983). Effect of hot preessing temperature on the thermal diffusivity/conductivity of SiC/AlN composites. J. Am. Ceram. Soc..

[24] Kim Y.W., Lim K.Y., Seo W.S. (2014). Microstructure and thermal conductivity of silicon carbide with yttria and Scandia. J Am. Ceram. Soc..

[25] Kim K.J., Kim Y.W., Lim K.Y., Nishimura T., Narimatsu E. (2015). Electrical and thermal properties of SiC-AlN ceramics without sintering additives. J. Eur. Ceram. Soc..

[26] Kingery W.D., Bowen H.K., Uhlmann D.R. (1976). Introduction to Ceramics.

